# Can dynamic light improve melatonin production and quality of sleep?

**DOI:** 10.1186/cc13204

**Published:** 2014-03-17

**Authors:** J Jennings, TD Thomsen, JW Larsen, J Markvardt

**Affiliations:** 1 Sheffield Teaching Hospital, Sheffield, UK; 2 Kolding Hospital, part of Lillebaelt Hospital, Kolding, Denmark; 3 Danish Building Research Institute/Aalborg University, Copenhagen, Denmark

## Introduction

Hospital lighting may cause disruption in the circadian rhythm, partly due to suppression of melatonin production. This may create sleep difficulties and delirium for ICU patients and health issues such as fatigue, poor sleep quality and chronic diseases for ICU staff. Dynamic RGB coloured light which changes colour and intensity in correlation with the time of day has been installed in a Danish ICU, aiming to create lighting conditions being close to daylight variations and supporting patient and staff rhythms. As the effect of dynamic light on patients may be biased by illness, organ failure, and so forth, the aim was to examine the influence of dynamic light on ICU nurses' melatonin production, quality of sleep, and well-being.

## Methods

An intervention study examining the impact of dynamic light regarding the impact on the circadian rhythm (measured by melatonin profiles from saliva samples), quality of sleep (sleep efficiency, number of awakenings and subjective assessment of sleep; measured by sleep monitors and sleep diary), and subjective experiences of well-being, health, and sleep quality (measured by a questionnaire survey). Results from the intervention group were compared with a control group of nurses from a similar ICU without dynamic light. Light conditions were documented by measurements. Data collection was from February to May 2013.

## Results

A total of 55 nurses (89%) from the intervention ICU (ICU1) and 58 nurses (88%) from the control ICU (ICU2) participated. No significant differences were found between the two groups regarding personal characteristics. The nurses from ICU1 described their work light as comfortable, relaxing and natural compared with artificial, institutional and gloomy in ICU2. Preliminary analyses did not shown any significant differences in melatonin level. During a 10-day period, the nurses from ICU2 assessed their actual sleep as less effective (OR 2.17; *P *= 0.03) and felt less rested (OR 1.89; *P *= 0.006) compared with nurses from ICU1. The nurses in ICU2 had 16% more awakenings (*P *= 0.05) during sleep, but there were no significant differences in duration of awakenings or in total sleep efficiency between the two groups.

## Conclusion

Most participants from the intervention ICU found the dynamic light agreeable and assessed their sleep more positively than participants from the control ICU. No significant differences were found between monitored sleep efficiency and melatonin level.

