# Erratum to: can dynamic light improve melatonin production and quality of sleep?

**DOI:** 10.1186/s13054-015-1016-4

**Published:** 2015-09-04

**Authors:** HI Jensen, TD Thomsen, JW Larsen, J Markvart

**Affiliations:** Kolding Hospital, part of Lillebaelt Hospital, Kolding, Denmark; Institute of Regional Health Research, University of Southern Denmark, Odense, Denmark; Danish Building Research Institute/Aalborg University, Copenhagen, Denmark

Unfortunately, the original version of this article [1] contained an error. The author’s names and affiliations have not been included correctly. The correct names and affiliations are:

HI Jensen^1,2*^, TD Thomsen^1^, JW Larsen^1^ and J Markvart^3^

* Corresponding author: Hanne Irene Jensen, Hanne.Irene.Jensen@rsyd.dk

^1^ Kolding Hospital, part of Lillebaelt Hospital, Kolding, Denmark

^2^ Institute of Regional Health Research, University of Southern Denmark

^3^ Danish Building Research Institute/Aalborg University, Copenhagen, Denmark
